# Antidepressant activity of anti-cytokine treatment: a systematic review and meta-analysis of clinical trials of chronic inflammatory conditions

**DOI:** 10.1038/mp.2016.167

**Published:** 2016-10-18

**Authors:** N Kappelmann, G Lewis, R Dantzer, P B Jones, G M Khandaker

**Affiliations:** 1 Department of Psychiatry, University of Cambridge, Cambridge, UK; 2 Division of Psychiatry, University College London, London, UK; 3 Department of Symptom Research, University of Texas MD Anderson Cancer Centre, Houston, TX, USA; 4 Cambridgeshire and Peterborough NHS Foundation Trust, Cambridge, UK

## Abstract

Inflammatory cytokines are commonly elevated in acute depression and are associated with resistance to monoaminergic treatment. To examine the potential role of cytokines in the pathogenesis and treatment of depression, we carried out a systematic review and meta-analysis of antidepressant activity of anti-cytokine treatment using clinical trials of chronic inflammatory conditions where depressive symptoms were measured as a secondary outcome. Systematic search of the PubMed, EMBASE, PsycINFO and Cochrane databases, search of reference lists and conference abstracts, followed by study selection process yielded 20 clinical trials. Random effect meta-analysis of seven randomised controlled trials (RCTs) involving 2370 participants showed a significant antidepressant effect of anti-cytokine treatment compared with placebo (standardised mean difference (SMD)=0.40, 95% confidence interval (CI), 0.22–0.59). Anti-tumour necrosis factor drugs were most commonly studied (five RCTs); SMD=0.33 (95% CI; 0.06–0.60). Separate meta-analyses of two RCTs of adjunctive treatment with anti-cytokine therapy and eight non-randomised and/or non-placebo studies yielded similar small-to-medium effect estimates favouring anti-cytokine therapy; SMD=0.19 (95% CI, 0.00–0.37) and 0.51 (95% CI, 0.34–0.67), respectively. Adalimumab, etanercept, infliximab and tocilizumab all showed statistically significant improvements in depressive symptoms. Meta-regression exploring predictors of response found that the antidepressant effect was associated with baseline symptom severity (*P*=0.018) but not with improvement in primary physical illness, sex, age or study duration. The findings indicate a potentially causal role for cytokines in depression and that cytokine modulators may be novel drugs for depression in chronically inflamed subjects. The field now requires RCTs of cytokine modulators using depression as the primary outcome in subjects with high inflammation who are free of other physical illnesses.

## Introduction

The association between the immune system and the brain may offer new mechanistic understanding and insights for novel therapies for depression. Cytokine-mediated communication between the immune system and the brain has been implicated in the pathogenesis of depression.^1, 2, 3^ Major depression is common (one in four) after interferon treatment, a potent inducer of cytokines, in patients affected by hepatitis C virus.^4^ Experimental immuno-activation in healthy volunteers leads to depressive symptoms and reduced cognitive performance.^5, 6^ Meta-analyses of cross-sectional studies confirm elevated levels of circulating inflammatory cytokines in depressed patients,^7, 8, 9, 10^ and longitudinal studies have demonstrated that elevated serum cytokine levels precede, so potentially cause depressive symptoms.^11, 12^ A dose–response relationship between serum concentration of interleukin 6 (IL-6) in childhood at 9 years and subsequent depressive symptoms in early-adulthood at 18 years has been reported in the Avon Longitudinal Study of Parents and Children birth cohort.^11^ A longitudinal association between circulating inflammatory markers and subsequent depressive symptoms has also been reported from the Whitehall II cohort.^12^ Furthermore, activation of the inflammatory system is thought to underlie antidepressant resistance highlighting an involvement in treatment response.^13, 14^ Therefore, whether targeting inflammation, particularly inflammatory cytokines, could provide therapeutic benefit for patients with depression is a key question.

A meta-analysis of randomised controlled trials (RCTs)^15^ of non-steroidal anti-inflammatory drugs (NSAIDs), given as sole treatment or as adjunct to antidepressants, indicates that they may be more effective than placebo in treating depression (Cohen’s *d*=0.27; 95% confidence interval (CI), 0.08–0.45),^16^ although there are limitations of individual studies included such as attrition. Although these results point to an inflammatory component to depression, further examination of the evidence regarding cytokine-modulating drugs may help to elucidate the relevance of inflammation for pathogenesis and treatment of depression specifically. NSAIDs are broad-spectrum anti-inflammatory agents that also act on other targets, such as glucocorticoid receptors.^17^ These receptors are, themselves, relevant for depression pathophysiology,^18^ so the extent to which the antidepressant effect of NSAIDs is due solely to anti-inflammatory action is unclear. Some NSAIDs such as cyclooxygenase-2 inhibitors increase risk of cardiovascular disease,^19^ a known comorbidity for depression, so their use in depression may be problematic. Besides, many trials of anti-inflammatory drugs for depression are based on people with chronic physical illness^16^ but it is unclear whether the improvement in depression is due to the improvement in physical illness.

Cytokine modulators, which include monoclonal antibodies and cytokine inhibitors, constitute a more pure anti-inflammatory class because they target-specific cytokine pathways. Recently, a proof-of-concept RCT of infliximab, a tumour necrosis factor alpha (TNF-α) specific monoclonal antibody, has reported improvements in patients with treatment-resistant depression characterised by high inflammation at baseline.^20^ Supported by a large number of RCTs, cytokine modulators are well established treatments for chronic inflammatory conditions such as rheumatoid arthritis^21^ and psoriasis.^22^ Many of these clinical trials have also reported on secondary psychosocial outcome measures including depressive symptoms, which could be utilised to address important questions regarding potential usefulness of anti-cytokine treatment for depression. In addition to quantifying the antidepressant effect of anti-cytokine treatment, examination of the relationship between the improvement in depressive symptoms and that in physical symptoms could elucidate the role of inflammation in depression specifically. Previous RCTs indicate baseline severity of depression moderates the antidepressant effects of monoaminergic drugs^23^ and psychotherapy;^24^ it not clear whether this is also the case for anti-cytokine treatment.

We report a systematic review and meta-analysis of secondary data from clinical trials of anti-cytokine treatment in chronic inflammatory conditions to address the following key outstanding questions: (1) does blocking-specific inflammatory cytokine pathways lead to improvement in depressive symptoms; (2) what is the relationship between the antidepressant effect and the improvement in physical illness; (3) is the antidepressant effect related to baseline severity of depressive symptoms; (4) is there any sex or age difference of the antidepressant effect. Answers to these questions would provide important clues on whether inflammatory cytokines have a causal role in depression, and whether cytokine modulators may be useful for treating depression.

## Materials and methods

### Search strategy and study selection

A systematic search of the PubMed, Embase, PsycINFO and Cochrane databases was carried out for all clinical trials of cytokine modulators involving human subjects published in the English language until 5th April 2016, where depression or depressive symptoms were reported as an outcome. The search terms included indexing terms as well as wildcards to maximise return: ‘(monoclonal antibod* OR cytokine inhibitor OR tocilizumab OR infliximab OR adalimumab OR ustekinumab OR etanercept OR dupilumab) AND (depression OR depressive symptom)’. We also searched reference lists of retrieved articles and conference abstracts, and wrote to key authors in the field for unpublished data.

Clinical trials of specific cytokine inhibitors or monoclonal antibodies against specific inflammatory cytokines that measured depressive symptoms using a recognised tool were included. In order to obtain a comprehensive overview of the literature, we included all clinical trial designs, that is, RCTs, double-blind or open-label, as well as non-randomised studies (analysed separately). Studies that examined cytokine modulators as an adjunct to interferon therapy, used monoclonal antibodies as a method for detecting leukocyte subtypes, or examined monoclonal antibodies against targets other than an inflammatory cytokine (for example, vascular growth factor, adhesion molecule) were excluded.^25^ The literature search and study selection were carried out by NK and GMK; any differences were resolved by discussion.

### Data extraction and statistical analysis

Data were extracted from published articles or obtained by directly contacting the authors if relevant data were not reported. When authors could not be reached but data were available in the paper in graph format (two RCTs),^26, 27^ values were extracted using software that has been shown to be a reliable method of data extraction for meta-analyses.^28^ RCTs were quality assessed using the CONSORT criteria;^29^ scores reflect the percentage of CONSORT items the study adhered to. We aligned depression scales so higher scores reflect higher symptom severity by multiplying scores from reversed scales by -1. For studies that included multiple intervention arms based on different dosages of the same drug, we pooled means and s.d.s of these groups to calculate a single outcome measure. Here, the pooled s.d. (sd_pooled_) equalled the square root of (sd_1_^2 × (n_1_−1)+sd_2_^2 × (n_2_−1))/((n_1_−1)+(n_2_−1)). Effect sizes were calculated as standardised mean difference (SMD) for continuous outcomes according to the formula 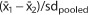. For binary outcomes odds ratios were created according to the formula (Outcome_1_Group_1_ × Outcome_2_Group_2_)/(Outcome_2_Group_1_ × Outcome_1_Group_2_). Then odds ratios were transformed to SMD by dividing the natural logarithm of the odds ratios by 1.81.^30^

Based on study methodology, three types of studies were combined separately using random effect meta-analysis: RCTs of anti-cytokine drug vs placebo; RCTs of adjunctive treatment with anti-cytokine therapy (anti-cytokine drug plus active treatment vs active treatment); and other trials (non-randomised and/or non-placebo studies). For RCTs effect estimates were calculated as SMD using depression severity scores in treatment and placebo groups at the end of trial. SMD is an appropriate measure of effect estimate when studies assess the same variable (for example, depressive symptoms) but measure it in a variety of ways.^31^ In addition, we calculated SMD using change in depression severity score from baseline to end of trial in each group to examine the robustness of original results (sensitivity analysis). Sensitivity analyses were also carried out after excluding specific RCTs from meta-analysis. For other trials, SMD was calculated as difference in depression severity score from baseline to end of trial, as no control group was present. All studies were weighted using an inverse-variance method so studies with larger samples were given higher weight. When possible, pooled effect estimates were calculated for specific drugs. Heterogeneity between studies was investigated by calculating the Cochrane’s heterogeneity statistic *Q* and the *I*^2^ statistic that represents the fraction of variation between studies attributable to heterogeneity.^32^ Publication bias was assessed for each group of studies by visual inspection of funnel plots including Egger’s test,^33^ and the trim and fill method.^34^ We analysed the RCTs of adjunctive treatment with anti-cytokine therapy separately because these studies compared combination of anti-cytokine drug and a disease-modifying anti-rheumatic drug (DMARD) with DMARD alone. One RCT^35^ administered adalimumab to all participants for 4 weeks before randomising them to placebo or adalimumab for subsequent 52 weeks, so we also included data from the first 4 weeks in the meta-analysis of ‘other trials’. Data from two studies^36, 37^ that randomised individuals to different dosage regimens of the same drug but lacked a placebo group, and a non-randomised study^38^ were also included in this category.

All secondary analyses were based on placebo-controlled RCTs only as they represent the highest quality of scientific evidence of efficacy. Meta-regressions were performed to examine the relationship between the effect estimates for depression and (1) the effect estimates for primary physical outcome; (2) baseline severity of depressive symptoms; (3) sex (percentage of male participants in each sample); and (4) mean age of sample. We quantified baseline depression symptom severity in a standardised manner rather than simply using mean scores because the studies used different scales to measure depression. We identified general population distributions for each scale^39, 40, 41, 42, 43^ that were used to calculate effect sizes (SMD) of baseline symptom severity for the sample compared with the general population. All analyses were performed in *R* using the *metaphor* package for meta-analysis and meta-regression;^44^ analysis codes are available on request.

## Results

**Figure 1:**
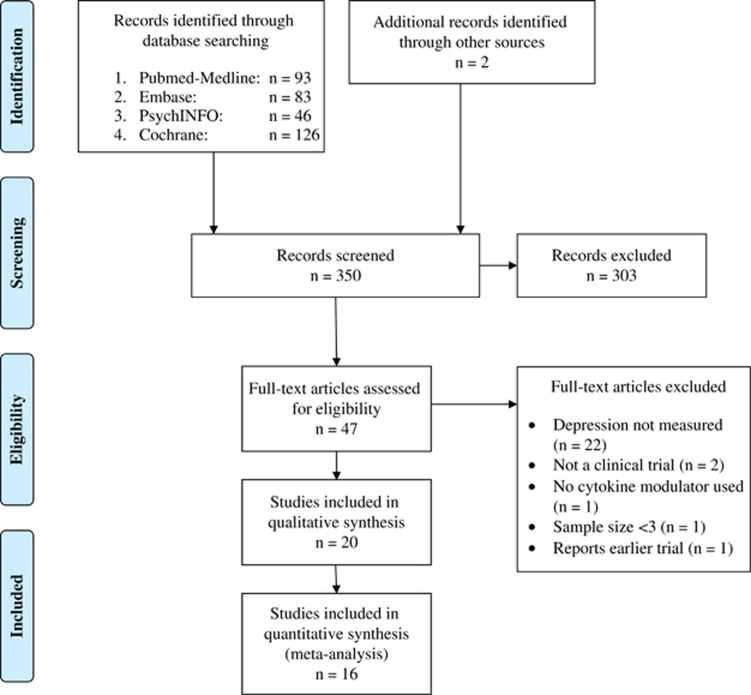
PRISMA Flow diagram of study selection for systematic review.

The literature search retrieved 350 potentially relevant articles of which 20 studies were finally included in the review (see Figure 1 for a PRISMA diagram of literature search). Of the included studies, seven were RCTs of anti-cytokine drug vs placebo, three were RCTs of adjunctive treatment with anti-cytokine therapy, and 10 were other study designs such as non-randomised and/or non-placebo studies (Table 1).

### Meta-analysis of RCTs of anti-cytokine drug vs placebo

**Figure 2:**
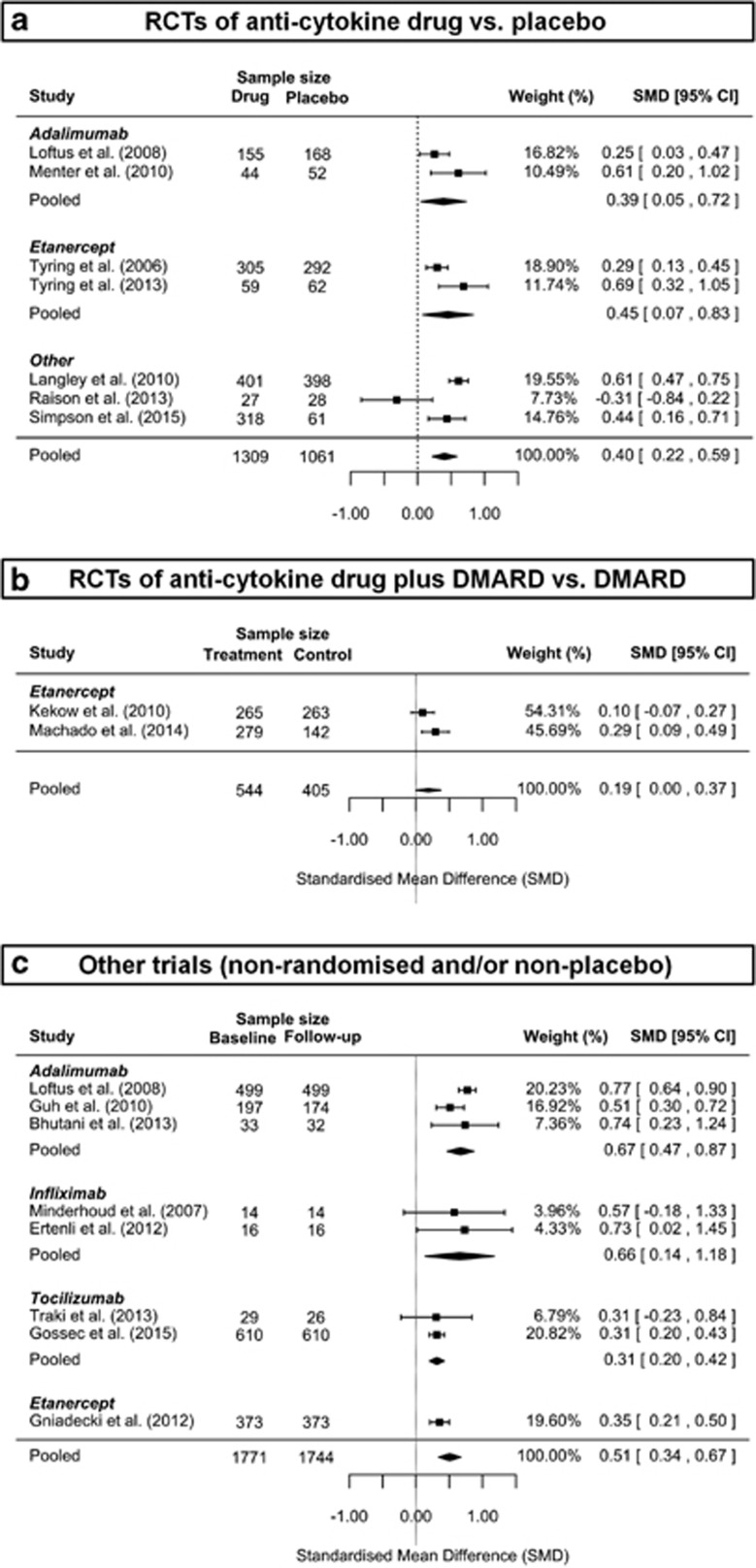
Meta-analysis of antidepressant activity of anti-cytokine treatment. (a) Meta-analysis of RCTs of anti-cytokine drug vs. placebo. (b) Meta-analysis of RCTs of anti-cytokine drug plus DMARD vs. DMARD. (c) Meta-analysis of other trials (non-randomised and/or non-placebo). CI, confidence interval; DMARD, Disease Modifying Anti-rheumatoid Drug; RCT, randomised controlled trial.

**Figure 3:**
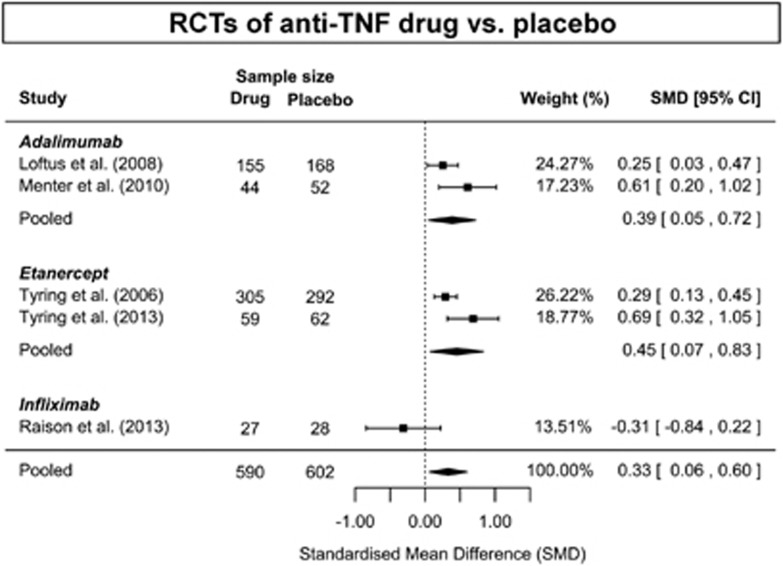
Antidepressant activity of anti-TNF treatment: meta-analysis of RCTs. CI, confidence interval; RCT, randomised controlled trial; TNF, tumor necrosis factor.

Meta-analysis of seven randomised, double-blind, placebo-controlled trials,^20, 26, 27, 35, 45, 46, 47^ involving 1309 subjects treated with anti-cytokine drugs and 1061 subjects treated with placebo, showed significant improvement in depressive symptoms with anti-cytokine treatment compared to placebo; SMD=0.40 (95% CI, 0.22–0.59) (Figure 2a). There was evidence of significant heterogeneity among studies (*P<*0.001; *I*^2^=73%). Anti-TNF drugs were most commonly studied (five RCTs),^20, 26, 27, 35, 46^ which as a group showed significant antidepressant effect; SMD=0.33; 95% CI, 0.06–0.60 (Figure 3). There was also evidence of significant heterogeneity among these studies (*P*=0.02; *I*^2^=75%). Regarding specific drugs, based on two trials each, the effect estimate for adalimumab^35, 46^ (SMD=0.39; 95% CI, 0.05–0.72) and that for etanercept^26, 27^ (SMD=0.45; 95% CI, 0.07–0.83) were similar (Figure 2a). There was no evidence of significant heterogeneity between the adalimumab trials (*P*=0.134; *I*^2^=55%) but there was evidence of heterogeneity among the etanercept trials (*P*=0.05, *I*^2^=73%).

Sensitivity analysis of seven RCTs,^20, 26, 27, 35, 45, 46, 47^ of anti-cytokine drugs vs placebo based on change in depression severity scores from baseline to end of trial yielded very similar results to that of the original analysis; SMD=0.37 (95% CI; 0.18–0.57) (see online Supplementary Figure 1). However, evidence for heterogeneity remained (*P*<0.001; *I*^2^=76%).

### Meta-analysis of RCTs of adjunctive treatment with anti-cytokine therapy

Of three trials^48, 49, 50^ that used anti-cytokine drugs as adjunctive treatment, two provided enough data for meta-analysis (Figure 2b).^49, 50^ Both of these studies compared combination of etanercept and a DMARD (*n*=544) with DMARD alone (*n*=405). Meta-analysis showed a small effect of etanercept plus DMARD on depressive symptoms compared with DMARD alone (SMD=0.19; 95% CI, 0.00–0.37). There was no evidence of heterogeneity between studies (*P*=0.158; *I*^2^=49%).

### Meta-analysis of other trials

Out of 10 non-randomised and/or non-placebo studies,^36, 37, 38, 51, 52, 53, 54, 55, 56, 57^ eight provided enough data for meta-analysis involving 1744 patients with data at baseline and follow-up (Figure 2c).^35, 36, 38, 51, 52, 53, 54, 55^ Meta-analysis of these studies suggested significant improvement in depressive symptoms following anti-cytokine treatment (SMD=0.51; 95% CI, 0.34–0.67). There was evidence of significant heterogeneity among these studies (*P*<0.001; *I*^2^=73%). Anti-TNF drugs were most commonly studied (six studies), which as a group showed significant antidepressant effect (SMD=0.58; 95% CI, 0.39–0.77) (see online Supplementary Figure 2). However, there was evidence for significant heterogeneity among these studies (*P*=0.002; *I*^2^=67%). Regarding specific drugs, adalimumab, infliximab and tocilizumab all showed statistically significant improvements in depressive symptoms. Meta-analytic effect estimates for adalimumab: SMD=0.67 (95% CI, 0.47–0.87); infliximab: SMD=0.66 (95% CI, 0.14–1.18); and tocilizumab: SMD=0.31 (95% CI, 0.20–0.42). There was no evidence of significant heterogeneity in any of these meta-analyses (all *P>*0.05).

### Association with improvement in physical illness

**Figure 4:**
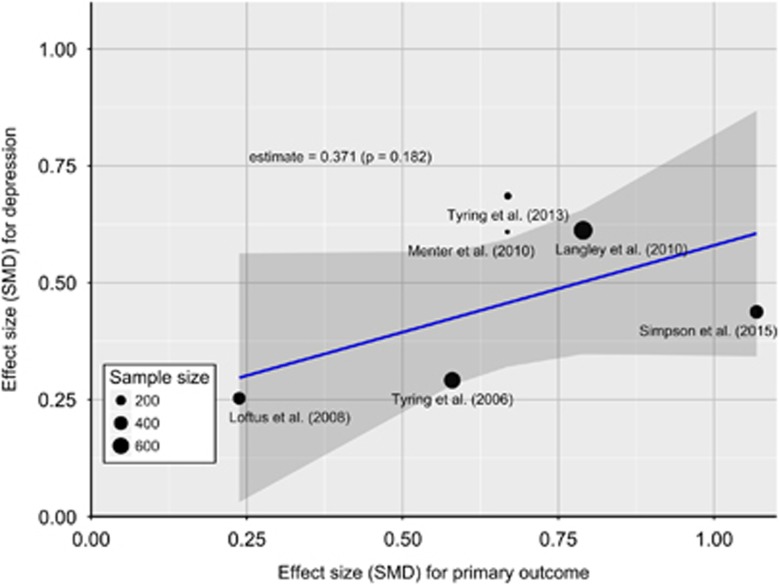
Meta-regression of the association between antidepressant effects and improvements in physical illness. *P-*value for meta-regression slope indicates no statistically significant association between antidepressant effect of anti-cytokine treatment and improvement in physical illness. The Figure shows that the antidepressant effect does not change significantly (that is, increase or decrease) across the range of effect estimates for physical illness. SMD, standardised mean difference.

Meta-regression of six RCTs^26, 27, 35, 45, 46, 47^ found no evidence for an association between improvement in depressive symptoms and that in primary physical illness outcome measure at the end of trial (slope=0.37; *SE*=0.28; *P*=0.182) (Figure 4). The study by Raison *et al.*^20^ was excluded from this analysis because in this trial depression was the primary outcome so no physical illness was studied.

### Association with depression symptom severity at baseline

Meta-regression of seven RCTs^20, 26, 27, 35, 45, 46, 47^ showed an association between antidepressant effect of anti-cytokine treatment and baseline severity of depressive symptoms (slope=−0.12; *SE*=0.05; *P*=0.018). However, the association became non-significant after excluding the RCT by Raison *et al.*^20^ (slope=−0.05; *SE*=0.12; *P*=0.662). We explored the association by excluding the RCT by Raison *et al.*^20^ because baseline depression severity was much higher in this study (SMD of baseline depression 6.36) compared with the other RCTs (0.08–1.91).

### Association with sex and age

There was no strong evidence for a sex difference in the antidepressant effect of anti-cytokine treatment as the association between improvements in depressive symptoms and the percentage of male subjects in the sample, based on meta-regression of seven RCTs,^20, 26, 27, 35, 45, 46, 47^ did not reach significance (slope=1.29; *SE*=0.66; *P*=0.051). Re-analysis after excluding the study by Raison *et al.*,^20^ an outlier in which only 33% of subjects were male, attenuated the association further (slope=0.66; *SE*=0.71; *P*=0.355). Similarly, there was no association between SMD for depressive symptoms and mean age of sample (slope=0.008; SE=0.028; *P*=0.788).

### Association with study duration

Duration of follow-up in the RCTs of anti-cytokine drug vs placebo was similar (12–16 weeks) except the study by Loftus *et al.* (Table 1). Meta-regression of seven RCTs^20, 26, 27, 35, 45, 46, 47^ showed no association between the antidepressant effect of anti-cytokine treatment and study duration (slope=−0.004; *SE*=0.007; *P*=0.52).

### Sensitivity analyses

Sensitivity analysis of RCTs^20, 35, 45, 46, 47^ excluding two studies by Tyring *et al.*^26, 27^ from which data were extracted using software did not change results substantially; SMD=0.37 (95% CI; 0.11–0.63) (see online Supplementary Figure 3). However, evidence for significant heterogeneity remained (*P*=0.003; *I*^2^=79%). Unlike the other RCTs, the trial by Raison *et al.*^20^ was based on cases of treatment-resistant depression without any significant physical comorbidity. Meta-analysis of RCTs^26, 27, 35, 45, 46, 47^ excluding this trial yielded a slightly larger effect estimate; SMD=0.46 (95% CI; 0.30–0.61) (see online Supplementary Figure 4). However, there was evidence for significant heterogeneity among these studies (*P*=0.016; *I*^2^=61%). The RCT by Loftus *et al.*^35^ was an outlier in terms of follow-up length (see Table 1). Meta-analysis excluding this RCT^20, 26, 27, 45, 46, 47^ did not alter results greatly; SMD=0.43 (95% CI; 0.21–0.65) (see online Supplementary Figure 5). However, evidence for significant heterogeneity remained (*P*=0.002; *I*^2^=76%).

### Assessment of publication bias

Visual inspection of funnel plot of effect estimates from seven RCTs suggested that there was no evidence of publication bias (see online Supplementary Figure 6), which was in line with a non-significant Egger’s test for funnel plot asymmetry (*P*>0.05). Similarly, trim and fill analyses did not show any evidence of funnel plot asymmetry for RCTs. There were too few studies to allow assessment of publication bias among RCTs of adjunctive treatment with anti-cytokine therapy. For the other trials (non-randomised, non-placebo) Egger’s test for funnel plot asymmetry (*P*>0.05) and visual inspection of the funnel plot did not suggest any evidence for publication bias (see online Supplementary Figure 7). However, the trim and fill method indicated presence of funnel plot asymmetry, and that additional four studies would be needed to reach symmetry leading to slight attenuation of effect size (trim and fill SMD=0.36; 95% CI, 0.18–0.55). It is known that the trim and fill method can underestimate effect estimates when there is significant heterogeneity among studies;^58^ indeed, after restricting the analysis to anti-TNF studies heterogeneity decreased and there was no longer any evidence of funnel plot asymmetry (*P*=0.50).

## Discussion

Findings from this large systematic review of 20 studies including meta-analyses of 16 studies totalling 5063 participants indicate that anti-cytokine treatment improves depressive symptoms. We observed significant results favouring cytokine modulators over respective control groups with effect estimates of 0.40 for RCTs of anti-cytokine treatment vs placebo, 0.19 for RCTs of adjunctive treatment with anti-cytokine therapy, and 0.51 for other (non-randomised and/or non-placebo) studies. Regarding predictors of response, additional analyses based on RCTs indicated that the antidepressant effect was associated with severity of depressive symptoms at baseline, but not with improvement in physical illness (primary outcome under investigation in all but one RCT), sex and age of participants, or study duration. Sensitivity analyses using a different method to calculate SMD or exclusion of specific RCTs did not alter results substantially suggesting that the findings are robust. The results provide important clues regarding the role of inflammatory cytokines in depression and the potential for cytokine modulators as treatments for depression. Anti-cytokine drugs seem to offer treatment effects in the range of small-to-moderate effect sizes, which is comparable to estimates observed for common antidepressants.^23^ The results are in line with a previous meta-analysis of NSAIDs, which included three RCTs of cytokine inhibitors.^16^ The results are also consistent with a recent meta-analysis of anti-TNF treatment in people with chronic physical illness that reported improvements in depression and anxiety symptoms.^59^ However, based on a meta-analysis of seven RCTs the current study provides a robust, statistically significant effect estimate favouring anti-cytokine treatment for depression. In addition to the randomised, double-blind, placebo-controlled clinical trials (gold standard), we have examined RCTs of adjunctive treatment with anti-cytokine therapy, and non-randomised studies offering a comprehensive update of the literature.

Studies included in this review except one by Raison *et al.*^20^ report continuous measures of depression, that is, symptom severity scores, not a categorical diagnosis of depression. To calculate effect size we have used depression severity scores for treatment and placebo groups at the end of trial for all RCTs including one by Raison *et al.* We also used change in depression scores from baseline to end of trial. The lack of an association between the improvement in depressive symptoms and that in primary physical illness points to a causal role for inflammatory cytokines in depression, suggesting that the mood improvement is not simply an artefact of feeling physically better after anti-cytokine treatment. Examination of the time course of effect in individual studies may provide some clues regarding the underlying mechanism of antidepressant effect of anti-cytokine treatment. The RCT by Tyring *et al.*^27^ reported scores for fatigue and depression during the course of 12-week treatment with etanercept vs placebo for psoriasis. Compared with placebo, etanercept led to improvements in fatigue by 2 weeks into the study and in depressive symptoms by week 4. In this study improvement in depressive symptoms was associated with improvement in fatigue but not (strongly) with improvement in psoriasis, which is in line with our meta-regression finding. Neurovegetative symptoms such as fatigue, which develop rapidly following immune activation in humans are attributed to actions of inflammatory cytokines on the brain.^60, 61^ Therefore, it is possible that the antidepressant effect of anti-cytokine treatment is mediated by improvements in cytokine-induced neurovegetative symptoms. This hypothesis needs testing in future studies.

In future, RCTs of anti-cytokine treatment using depression as the primary outcome in subjects with high inflammation who are free of other physical illnesses are needed. Although further studies are currently underway (for example, NCT02363738 and NCT02473289), results from the RCT by Raison *et al.*^20^ included in this meta-analysis showed beneficial effects of infliximab for treatment-resistant depression cases only in those with elevated serum CRP levels at baseline. Treatment-resistant depression cases with elevated inflammatory marker levels may be ideal candidates for RCTs of cytokine modulators for a number of reasons. About a third of all depressed patients are antidepressant resistant^62^ and about a third of all depressed patients have elevated serum CRP (>3 mg l^−1^).^63^ This may not be a coincidence. Indeed, activation of the inflammatory system as reflected by elevated serum inflammatory marker concentrations predicts poor antidepressant response,^13, 14^ and treatment-resistant patients continue to show elevated cytokine levels.^64, 65^ Inflammation and consequent activation of the tryptophan-metabolising enzyme indoleamine 2,3-dioxygenase is thought to underlie persistent symptoms despite antidepressant therapy in depressed patients who are ‘inflamed’.^66^ Inflammatory cytokines such as interferon-γ, TNF-α and IL-6 can induce indoleamine 2,3-dioxygenase.^67, 68^ In mice, blocking TNF-α with etanercept^67^ or IL-6 with a monoclonal antibody^69^ have been reported to prevent depression-like behaviour following exposures to an inflammatory stimulus or stress. Therefore, cytokine-modulating therapy is likely to be beneficial for a subset of depressed patients, specifically those with evidence of inflammation.

Regarding specific types of studies, meta-analysis of two RCTs of anti-cytokine drugs as adjuncts to DMARD treatment showed a smaller effect size than that of the placebo-controlled randomised trials, which might be due to potential antidepressant effects of drugs used as comparison treatment. On the other hand, the pooled effect size for the non-randomised and/or non-placebo studies was larger than that for RCTs probably because of the lack of a comparison group to account for placebo effect. The association between baseline severity of depressive symptoms and response to anti-cytokine treatment was markedly reduced after excluding an outlier although an association between initial severity and antidepressant response is well known.^23^ This is possibly because depression severity was relatively low in all of the RCTs except the one by Raison *et al.,*^20^ which included cases of treatment-resistant depression. It is well known that baseline symptom severity is positively correlated with antidepressant treatment effect, so demonstration of a beneficial effect of anti-cytokine treatment in samples with relatively low depression scores indicates strong antidepressant properties of these drugs. There was no evidence for an association between antidepressant effect of cytokine modulators and sex or age of participants. Similar to depression, autoimmune chronic inflammatory conditions such as rheumatoid arthritis are more common in women than men (3:1),^70^ but sex does not influence response to anti-TNF therapy in patients with established rheumatoid arthritis,^71^ which is in line with our findings. A possible explanation for the lack of an association with age could be limited variability in age among the included studies (<10 years).

Strengths of the systematic review presented here include a relatively large number of studies including RCTs, which were quality assessed. The literature search was comprehensive, as supported by a lack of evidence for publication bias for most analyses. Inclusion of different trial methodologies allowed meta-analyses of two types of RCTs and non-randomised studies. The focus on a specific type of anti-inflammatory drug (that is, cytokine modulators) helped to demonstrate the relevance of inflammation, particularly inflammatory cytokines, for the pathogenesis and treatment of depression. A potential limitation is the use of data extracted from published graphs due to lack of response from authors of two studies.^26, 27^ However, the software used to extract data has been reported to be a reliable method for data extraction for meta-analysis,^28^ so any variation from original data would be too small to have any meaningful impact on the pooled effect estimate. Because a physical illness was the primary outcome in all but one studies included in this review, we were not able to comment on potential side-effects of cytokine-modulating treatment in depressed individuals specifically. Administration of monoclonal antibodies carries the risk of immune reactions such as acute anaphylaxis as well as various target-specific adverse effects including increased risk of infections, cancer and autoimmune disease.^72^ Infliximab and placebo groups in the study by Raison *et al.*^20^ were similar in terms of adverse events (except increased urinary leukocyte esterase in the placebo group) and no serious side-effects were observed in either group. Nevertheless, further studies regarding safety and tolerability of cytokine modulators in depressed individuals are needed. There was evidence for significant heterogeneity in the meta-analysis of RCTs and that of other trials. Heterogeneity is likely to be driven by heterogeneity of effect size because all studies included in these analyses except one showed effect in the same direction, that is, anti-cytokine treatment improves depressive symptoms (see Figures 2a and c). Heterogeneity was reduced when studies were grouped by specific monoclonal antibodies. For example, there was no evidence of heterogeneity among studies of adalimumab, infliximab and tocilizumab. However, sensitivity analyses after excluding specific RCTs did not reduce heterogeneity significantly.

In conclusion, this systematic review and meta-analysis provides an up-to-date summary of the existing literature on antidepressant effect of anti-cytokine treatment. The findings show robust improvements in depressive symptoms after anti-cytokine therapy (monoclonal antibody or cytokine inhibitor) with a small-to-moderate size effect. These results suggest inflammatory cytokines may have a key role in the pathogenesis of depression and that anti-cytokine drugs may be effective for some patients with depression, particularly treatment-resistant cases characterised by increased inflammation. The field now needs RCTs of anti-cytokine treatment using such patients, which would pave the way for novel, effective and personalised treatment for depression and could reduce the burden presented by such a serious and multifactorial illness.

## Figures and Tables

**Table 1:** Clinical trials included in the systematic review of antidepressant activity of anti-cytokine treatment

*Study*	*Design*	*Drug (*n*)*	*Placebo/comparison group (*n*)*	*Main outcome*	*Drug target*	*Dosage*	*Study duration*	*Assessment of depressive symptoms*	*Change in mean depression score from baseline*^a^		*CONSORT score (%)*
									*Treatment group*	*Placebo/comparison group*	
*RCTs of anti-cytokine drug* vs *placebo*											
Tyring *et al.* (2006)^27^	Double-blind RCT	Etanercept (305)	Placebo (292)	Psoriasis	TNF-α	50 mg twice weekly	12 weeks	BDI	−3.9	−2.1	94%
Loftus *et al.* (2008)^35^ (weeks 4–56)	Double-blind RCT	Adalimumab (324)	Placebo (168)	Crohn’s Disease	TNF-α	40 mg weekly or every other week	52 weeks	ZDS	−1.1	+1.8	53%
Langley et al. (2010)^47^	Double-blind RCT	Ustekinumab (800)	Placebo (398)	Psoriasis	IL-12, 23	45/90 mg at weeks 0,4; then every 12 weeks	12 weeks	HADS-D	−1.9	+0.2	52%
Menter *et al.* (2010)^46^	Double-blind RCT	Adalimumab (44)	Placebo (52)	Psoriasis	TNF-α	40 mg every other week	12 weeks	ZDS	−6.7	−1.6	56%
Tyring *et al.* (2013)^26^	Double-blind RCT	Etanercept (59)	Placebo (62)	Psoriasis	TNF-α	50 mg twice weekly	12 weeks	PROMIS depression score	−5.5	−2.7	b
Raison *et al.* (2013)^20^	Double-blind RCT	Infliximab (27)	Placebo (28)	Treatment-resistant depression	TNF-α	5 mg kg^−1^ at weeks 0, 2 and 6	12 weeks	HAM-D	−7.5	−9.6	91%
Simpson *et al.* (2015)^45^	Double-blind RCT	Dupilumab (318)	Placebo (61)	Atopic dermatitis	IL-4 R-α	Variable (100 to 600 mgs every 1–4 weeks)	16 weeks	HADS	−4.2	+0.1	b

*RCTs of anti-cytokine drugs as adjuncts to an active treatment*											
Kekow *et al.* (2010)^50^	Double-blind RCT	Etanercept+DMARD (265)	DMARD (263)	Rheumatoid arthritis	TNF-α	50 mg weekly	52 weeks	HADS-D	−2.4	−2.0	48%
Bae *et al.* (2013)^48^	Randomised open-label	Etanercept+DMARD (192)	DMARD (100)	Rheumatoid arthritis	TNF-α	25 mg twice weekly	16 weeks	HADS-D	−2.2	−1.3	63%
Machado *et al.* (2014)^49^	Randomised open-label	Etanercept+DMARD (279)	DMARD (142)	Rheumatoid arthritis	TNF-α	50 weekly	24 weeks	HADS-D	−2.8	−1.9	77%

*Non-randomised and/or non-placebo trials*											
Minderhoud *et al.* (2007)^38^	Single-blind	Infliximab (14)	—	Crohn’s Disease	TNF-α	5 mg kg^−1^ at baseline	4 weeks	CES-D	−5.7	—	—
Loftus *et al.* (2008)^35^ (weeks 0–4)	Open-label	Adalimumab (499)	—	—	TNF-α	80 mg at baseline; 40 mg at week 2	4 weeks	ZDS	−9.1	—	—
Gelfand *et al.* (2008)^56^	Open-label	Etanercept (2546)	—	—	TNF-α	50 mg twice weekly	12 weeks	BDI	Not reported	—	—
Dauden *et al.* (2009)^37^	Randomised (to dosage regimen), open-label	Etanercept (703)	—	—	TNF-α	25 or 50 mg twice weekly	54 weeks	HADS-D	−1.6	—	—
Guh *et al.* (2010)^51^	Open-label	Adalimumab (174)	—	—	TNF-α	80 mg at baseline; 40 mg every other week	24 weeks	BDI	−4.4	—	—
Ertenli *et al.* (2012)^53^	Open-label	Infliximab (16)	—	—	TNF-α	5 mg kg^−1^ at weeks 0, 2 and 6	6 weeks	HADS-D	−3.0	—	—
Gniadecki *et al.* (2012)^36^	Randomised (to dosage regimen) double-blind	Etanercept (752)	—	—	TNF-α	50 mg weekly; 50 mg twice weekly	12 weeks	HADS-D	−1.5	—	—
Bhutani *et al.* (2013)^52^	Open-label	Adalimumab (32)	—	—	TNF-α	80–40 mg every other week	24 weeks	PGWB depression	−1.9	—	—
Eisenberg *et al.* (2013)^57^	Open-label	Eisenberg (9)	—	Complex regional pain syndrome type 1	TNF-α	40 mg twice every other week	4 weeks	BDI	Not reported	—	—
Traki *et al.* (2013)^54^	Open-label	Tocilizumab (26)	—	Rheumatoid arthritis	IL-6 R	8 mg kg^−1^ monthly	26 weeks	HADS-D	−1.0	—	—
Gossec *et al.* (2015)^55^	Open-label	Tocilizumab (610)	—	Rheumatoid arthritis	IL-6 R	–	–	HADS-D	−1.3	—	—

Abbreviations: BDI, Beck’s Depression Inventory; CES-D, Centre for Epidemiological Studies Depression Scale; DMARD, disease-modifying anti-rheumatic drug; HADS(-D), Hospital Anxiety and Depression Scale (Depression); HAM-D, Hamilton Depression Rating Scale; IL, interleukin; PGWB depression, Psychological General Well-Being depression; RCT, randomised controlled trial; TNF-α, tumor necrosis factor alpha; ZDS, Zung Depression Inventory.

a (−)ve=decrease in depression score from baseline, (+)ve=increase in depression score from baseline.

b Could not assess fully because articles published as a letter or as a conference abstract (data from Tyring *et al.* was extracted using software and from Simpson *et al.* by contacting authors directly).
