# Supplementary material for: Antidepressant activity of anti-cytokine treatment: a systematic review and meta-analysis of clinical trials of chronic inflammatory conditions
Source: Mol Psychiatry. 2016 Oct 18;23(2):335–43. doi: 10.1038/mp.2016.167 (PMC5794896; doi:10.1038/mp.2016.167)
Supplement: Supplementary Figures [file mp2016167x1.doc]

**Online Supplementary Material**

Online Supplementary Figure 1: Meta-analysis of RCTs Using Change in Depression Score from Baseline to End of Trial


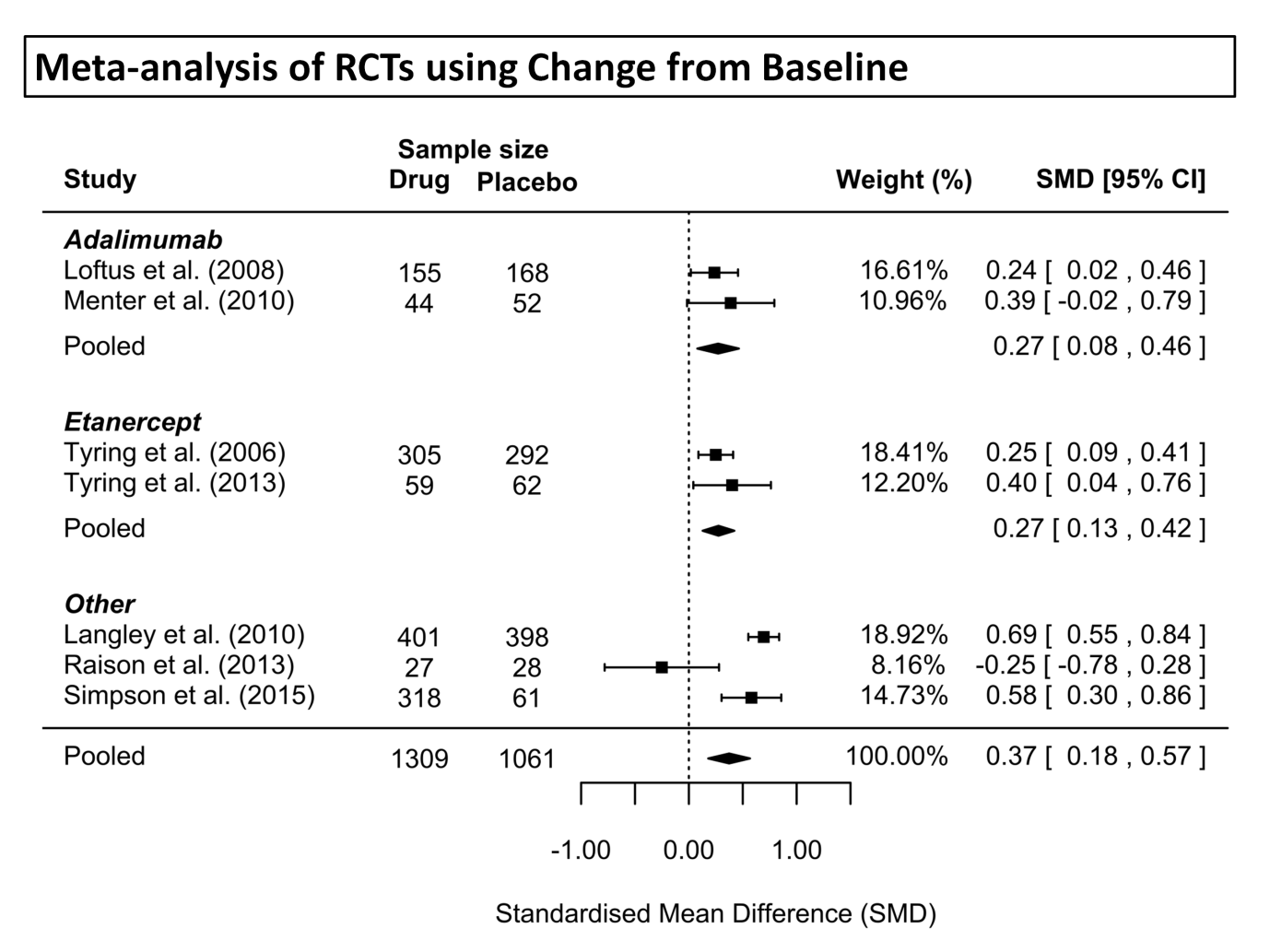


Online Supplementary Figure 2: Meta-analysis of non-randomised and/or non-placebo studies of anti-TNF treatment


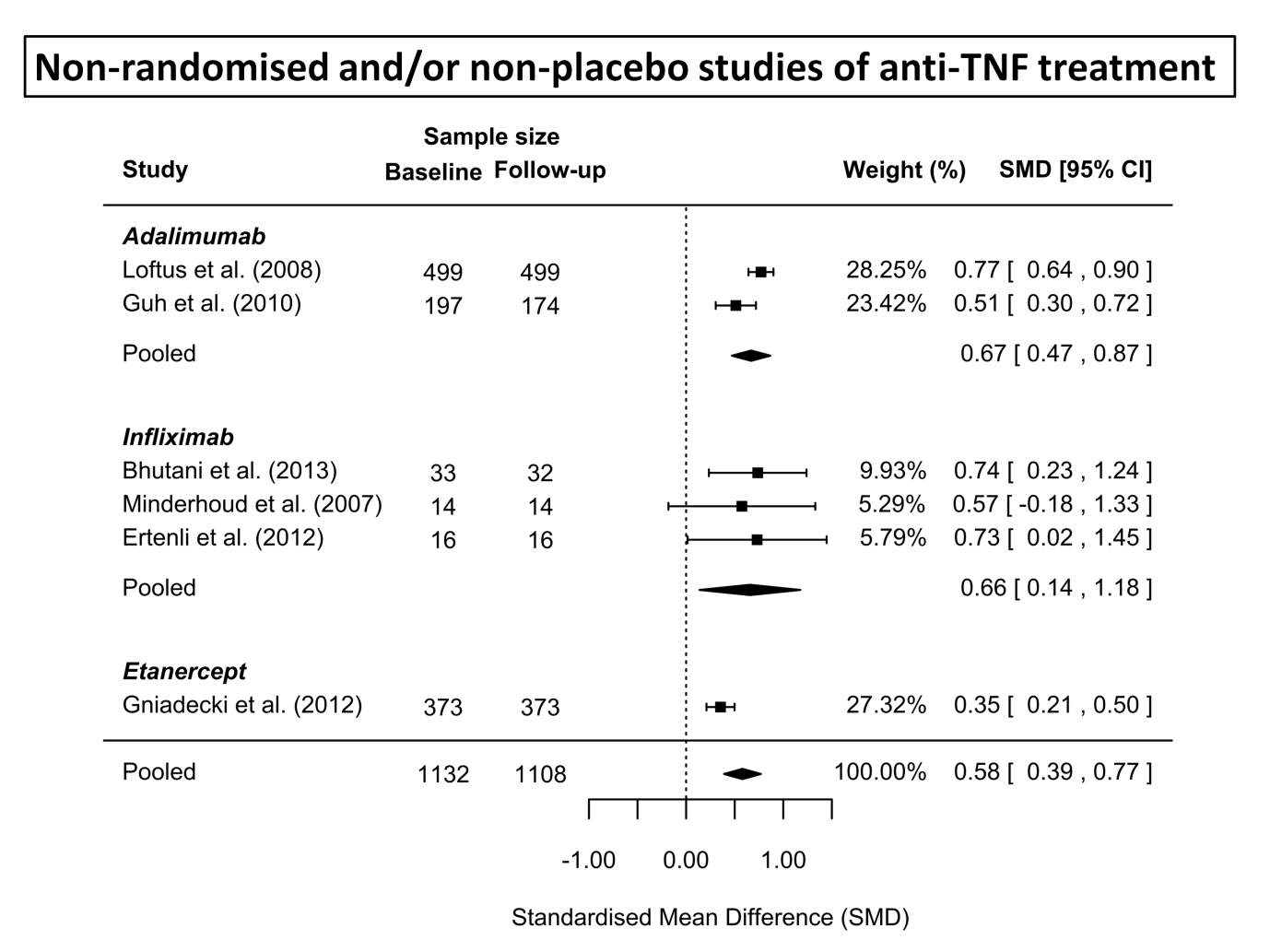


Online Supplementary Figure 3: Meta-analysis of RCTs after excluding two studies (Tyring 2006 and Tyring 2013) from which data were extracted using software


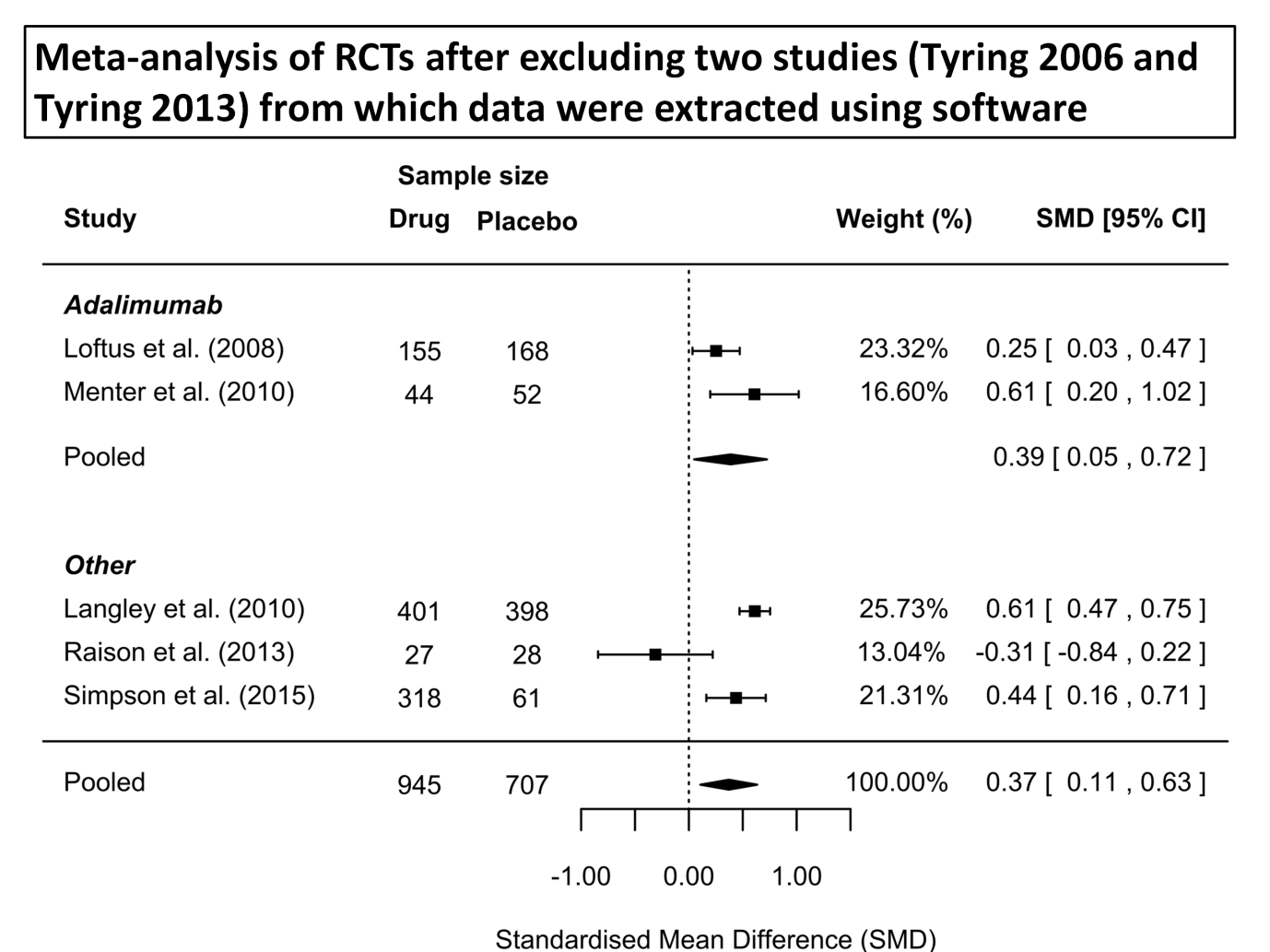


Online Supplementary Figure 4: Meta-analysis of RCTs after excluding Raison et al.


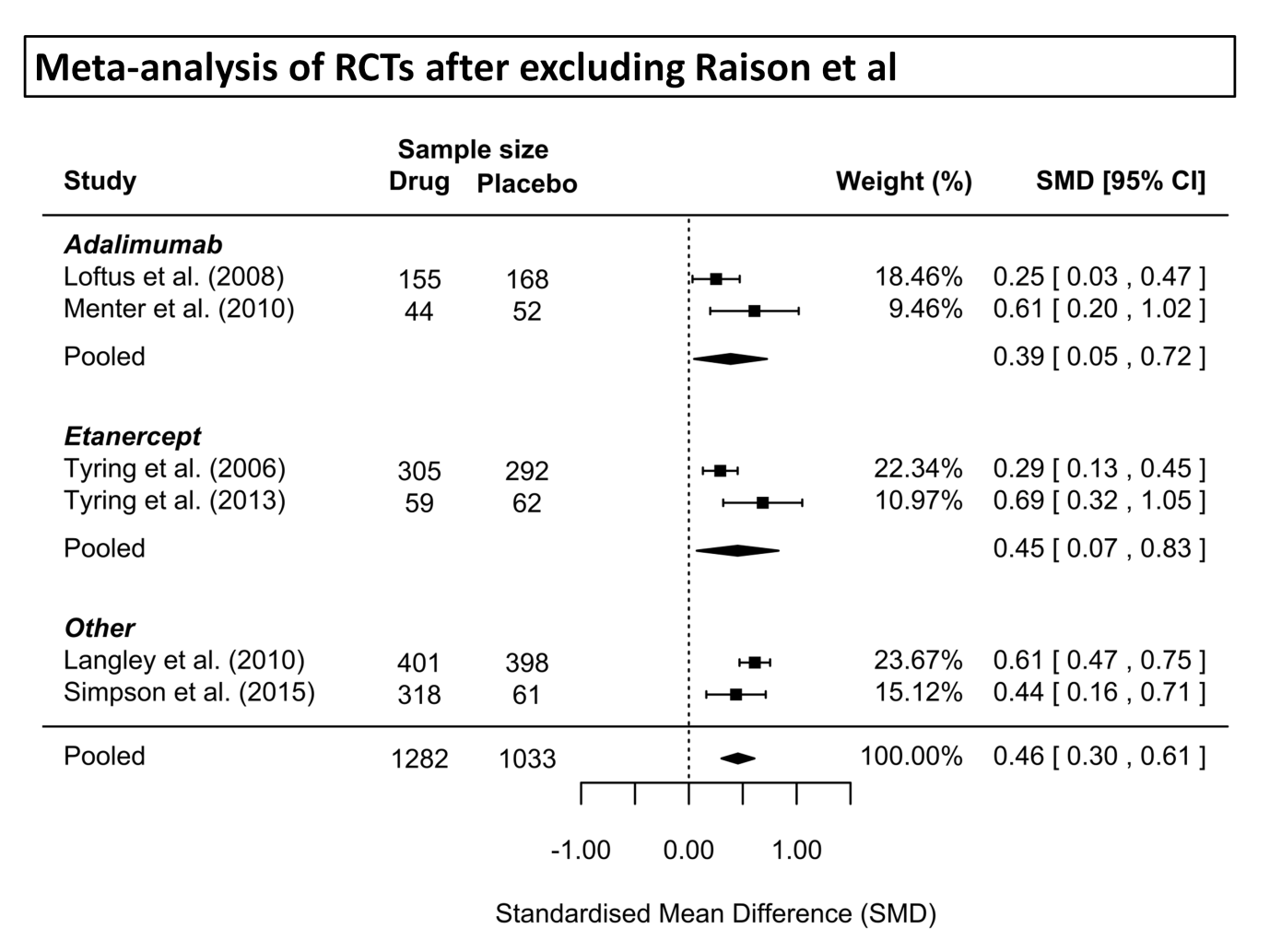


Online Supplementary Figure 5: Meta-analysis of RCTs after excluding Loftus et al.


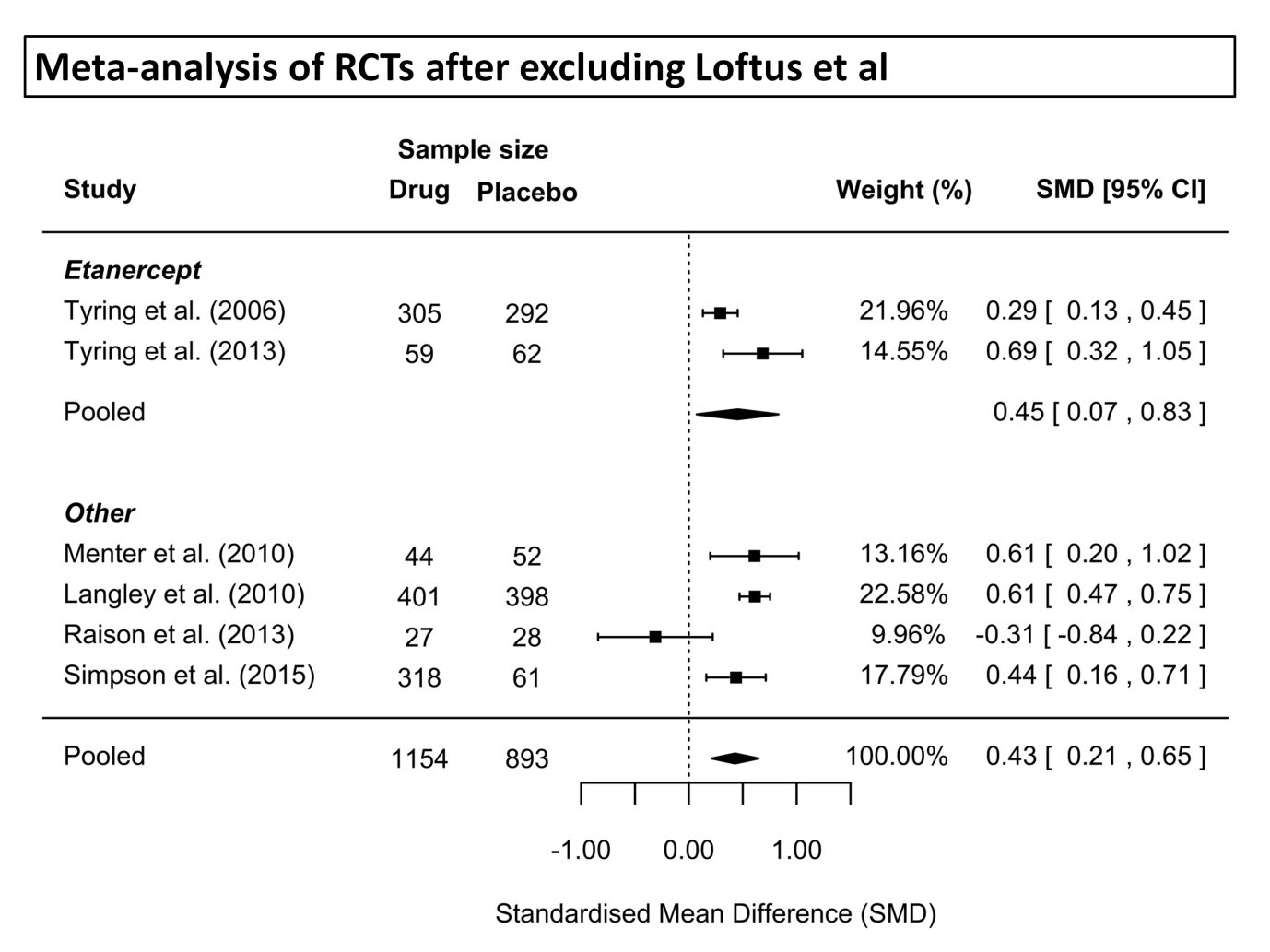


Online Supplementary Figure 6: Funnel Plot of RCTs of Anti-cytokine Drug vs. Placebo


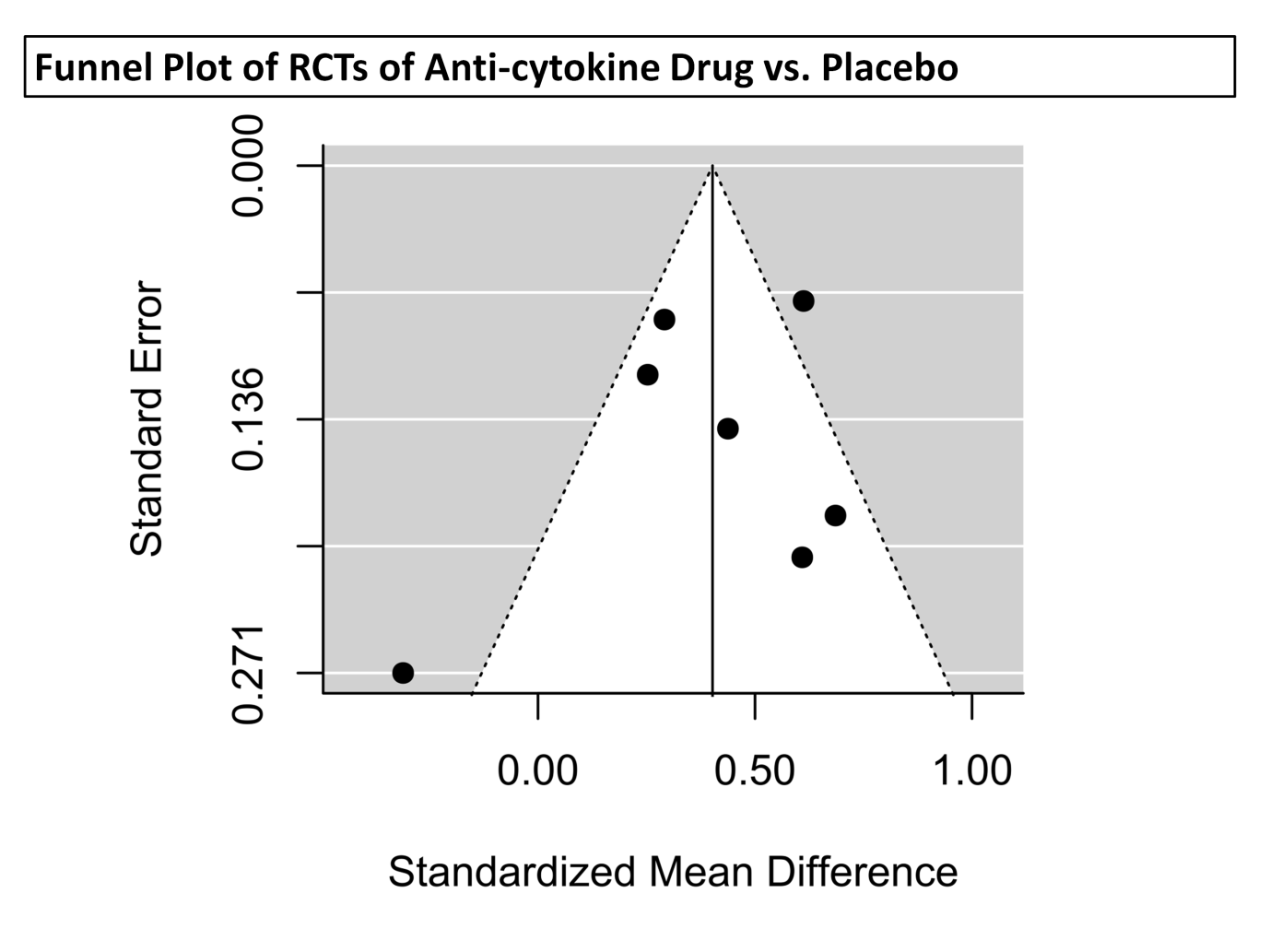


Online Supplementary Figure 7: Funnel Plot of Non-randomized and/or Non-placebo Studies

**
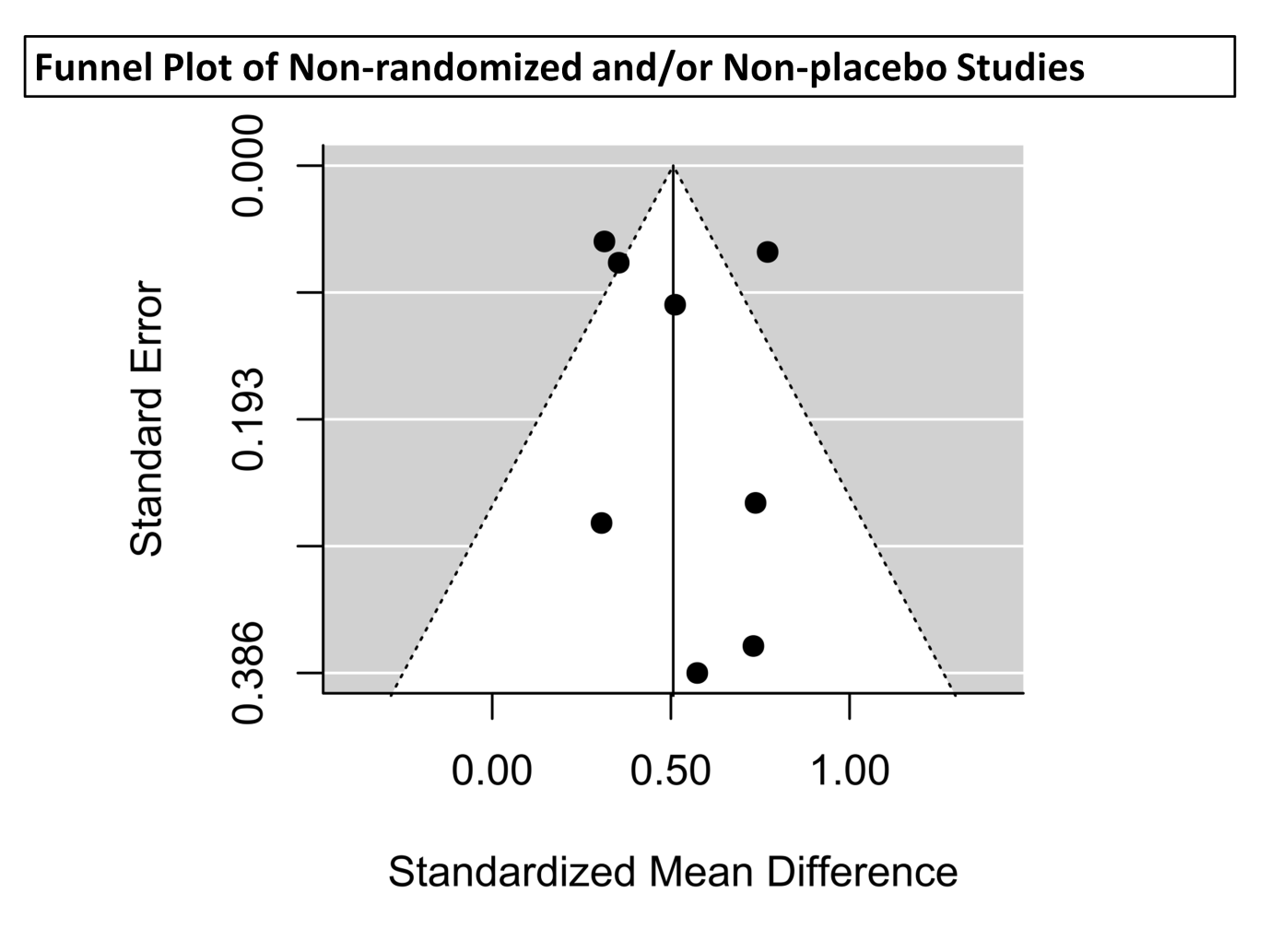
**
